# Supplementary material for: Heat adaptation of phage T7 under an extended genetic code
Source: Virus Evol. 2021 Dec 1;7(2):veab100. doi: 10.1093/ve/veab100 (PMC8923235; doi:10.1093/ve/veab100)
Supplement: veab100_Supp [file veab100_supp.zip › Sequencing_Analysis.zip!/Sequencing_Analysis/A2/marginal.html]

BRESEQ :: Marginal Predictions


|  |  |
| --- | --- |
|  | ***breseq***  version 0.30.1    mutation predictions \| marginal predictions \| summary statistics \| genome diff \| command line log |

| Marginal read alignment evidence | | | | | | | | | | | |
| --- | --- | --- | --- | --- | --- | --- | --- | --- | --- | --- | --- |
|  | seq id | position | | ref | new | freq | score (cons/poly) | reads | annotation | genes | product |
| \* | NC\_001604.1 | 35,366 | 0 | C | A | 68.4% | 153.2 / inf | 537 | intergenic (–/–) | *–/–* | –/– |
| \* | NC\_001604.1 | 27,783 | 0 | G | A | 67.7% | 397.2 / inf | 510 | intergenic (–/–) | *–/–* | –/– |
| \* | NC\_001604.1 | 33,019 | 0 | G | C | 48.8% | 53.1 / inf | 496 | intergenic (–/–) | *–/–* | –/– |
| \* | NC\_001604.1 | 35,266 | 0 | A | G | 41.1% | 126.3 / inf | 448 | intergenic (–/–) | *–/–* | –/– |
| \* | NC\_001604.1 | 26,933 | 0 | A | G | 36.7% | 217.8 / inf | 411 | intergenic (–/–) | *–/–* | –/– |
| \* | NC\_001604.1 | 3,371 | 0 | C | T | 31.8% | 479.6 / 213.0 | 380 | intergenic (–/–) | *–/–* | –/– |
| \* | NC\_001604.1 | 35,345 | 0 | C | A | 29.2% | 980.0 / 180.8 | 515 | intergenic (–/–) | *–/–* | –/– |
| \* | NC\_001604.1 | 35,254 | 0 | A | G | 27.7% | 465.5 / 262.1 | 445 | intergenic (–/–) | *–/–* | –/– |
| \* | NC\_001604.1 | 32,870 | 0 | G | T | 20.5% | 977.7 / 85.1 | 405 | intergenic (–/–) | *–/–* | –/– |

| Marginal new junction evidence (sorted from low to high skew) | | | | | | | | | | | |
| --- | --- | --- | --- | --- | --- | --- | --- | --- | --- | --- | --- |
|  | | seq id | position | reads (cov) | reads (cov) | score | skew | freq | annotation | gene | product |
| \* | ? | NC\_001604.1 | = 16476 | 362 (0.790) | 39 (0.090) | 31/590 | 11.1 | 9.2% | intergenic (–/–) | *–/–* | –/– |
|  | ? | NC\_001604.1 | 17143 = | 418 (0.930) |  |  |  |  | intergenic (–/–) | *–/–* | –/– |
| \* | ? | NC\_001604.1 | = 16604 | 373 (0.820) | 30 (0.070) | 26/590 | 12.4 | 6.9% | intergenic (–/–) | *–/–* | –/– |
|  | ? | NC\_001604.1 | 17028 = | 438 (0.980) |  |  |  |  | intergenic (–/–) | *–/–* | –/– |
| \* | ? | NC\_001604.1 | = 7781 | 452 (0.990) | 10 (0.020) | 7/558 | 19.9 | 2.3% | intergenic (–/–) | *–/–* | –/– |
|  | ? | NC\_001604.1 | 7899 = | 446 (1.050) |  |  |  |  | intergenic (–/–) | *–/–* | –/– |
| \* | ? | NC\_001604.1 | = 8253 | 443 (0.970) | 7 (0.020) | 7/584 | 20.4 | 1.4% | intergenic (–/–) | *–/–* | –/– |
|  | ? | NC\_001604.1 | 8503 = | 536 (1.210) |  |  |  |  | intergenic (–/–) | *–/–* | –/– |
| \* | ? | NC\_001604.1 | = 13578 | 0 (0.000) | 7 (0.020) | 6/584 | 21.0 | 24.1% | intergenic (–/–) | *–/–* | –/– |
|  | ? | NC\_001604.1 | 14348 = | 44 (0.100) |  |  |  |  | intergenic (–/–) | *–/–* | –/– |
| \* | ? | NC\_001604.1 | = 13467 | 2 (0.000) | 7 (0.020) | 6/592 | 21.2 | 82.5% | intergenic (–/–) | *–/–* | –/– |
|  | ? | NC\_001604.1 | 14266 = | 1 (0.000) |  |  |  |  | intergenic (–/–) | *–/–* | –/– |
| \* | ? | NC\_001604.1 | = 13268 | 206 (0.450) | 5 (0.010) | 4/588 | 22.6 | 4.7% | intergenic (–/–) | *–/–* | –/– |
|  | ? | NC\_001604.1 | 14300 = | 1 (0.000) |  |  |  |  | intergenic (–/–) | *–/–* | –/– |
| \* | ? | NC\_001604.1 | = 7605 | 543 (1.190) | 8 (0.020) | 3/578 | 23.3 | 1.5% | intergenic (–/–) | *–/–* | –/– |
|  | ? | NC\_001604.1 | 7904 = | 504 (1.150) |  |  |  |  | intergenic (–/–) | *–/–* | –/– |
| \* | ? | NC\_001604.1 | = 1252 | 0 (0.000) | 3 (0.010) | 3/580 | 23.4 | 100% | intergenic (–/–) | *–/–* | –/– |
|  | ? | NC\_001604.1 | 2742 = | 0 (0.000) |  |  |  |  | intergenic (–/–) | *–/–* | –/– |
| \* | ? | NC\_001604.1 | = 7613 | 539 (1.180) | 7 (0.020) | 3/586 | 23.5 | 1.3% | intergenic (–/–) | *–/–* | –/– |
|  | ? | NC\_001604.1 | 7912 = | 522 (1.170) |  |  |  |  | intergenic (–/–) | *–/–* | –/– |
